# Supplementary material for: Gaming in Pandemic Times: An International Survey Assessing the Effects of COVID-19 Lockdowns on Young Video Gamers’ Health
Source: Int J Environ Res Public Health. 2023 Sep 28;20(19):6855. doi: 10.3390/ijerph20196855 (PMC10572799; doi:10.3390/ijerph20196855)
Supplement: Supplementary file 1 [file ijerph-20-06855-s001.zip › ijerph-2579457-supplementary.pdf]

Covid-19 and esports: How the context of a global pandemic affected the lifestyle in gamers

This survey is distributed by a group of researchers from multiple universities. Our main goal is to better understand the lifestyle patterns of gamers and the challenges that they face during a global pandemic such as that of COVID-19. We very much appreciate your participation. By answering this survey and sharing it with your fellow gamers you will be helping the gaming community. Number of questions: 35 Completion time ~ 5min Please note that this is an anonymous survey. There are no questions that can identify who you are and we are unable to contact you following this survey. Please read below:Confidentiality:

Research records and data will be stored and maintained by New York Institute of Technology (NYIT) using Research Electronic Data Capture (REDCap) software under license from Vanderbilt University. REDCap is a secure web application for building and managing online surveys and databases. At NYIT, REDCap is installed on-premises on a web server located behind a firewall augmented by an Intrusion Preventions System (IPS). The Institute's REDCap database is stored and maintained on a different server with additional firewall protections. Access to the web and database servers is restricted to the NYIT Information Technology Department System Administration staff and to select members of the support staff of NYIT's College of Osteopathic Medicine. All reasonable efforts have been and will be made to keep your personal information confidential. However, total confidentiality cannot be guaranteed.

Please complete the survey below.

Thank you!

---

1) What is your age?

- ◯ 18-20
- ◯ 21-25
- ◯ 26-35
- ◯ 36-45
- ◯ more than 45

---

2) What do you identify as?

- ◯ Man
- ◯ Woman
- ◯ Non-binary
- ◯ Prefer not to disclose

---

3) Please specify your ethnicity.

- ◯ Hispanic or Latino
- ◯ African American or Black
- ◯ White
- ◯ Native American or American Indian
- ◯ Asian/Pacific Islander
- ◯ Prefer not to say

---

4) What is your educational level?

- ◯ High-School / Secondary
- ◯ College / Undergraduate / CEGEP
- ◯ Graduate (Master / PhD)
- ◯ Other

5) Where do you currently reside?

- ◯ Afghanistan
- ◯ Albania
- ◯ Algeria
- ◯ Andorra
- ◯ Angola
- ◯ Antigua and Barbuda
- ◯ Argentina
- ◯ Armenia
- ◯ Aruba
- ◯ Australia
- ◯ Austria
- ◯ Azerbaijan
- ◯ The
- ◯ Bahrain
- ◯ Bangladesh
- ◯ Barbados
- ◯ Belarus
- ◯ Belgium
- ◯ Belize
- ◯ Benin
- ◯ Bhutan
- ◯ Bolivia
- ◯ Bosnia and Herzegovina
- ◯ Botswana
- ◯ Brazil
- ◯ Brunei
- ◯ Bulgaria
- ◯ Burkina Faso
- ◯ Burma
- ◯ Burundi
- ◯ Cambodia
- ◯ Cameroon
- ◯ Canada
- ◯ Cape Verde
- ◯ Central African Republic
- ◯ Chad
- ◯ Chile
- ◯ China
- ◯ Colombia
- ◯ Comoros
- ◯ Republic of the
- ◯ Costa Rica
- ◯ Cote d'Ivoire
- ◯ Croatia
- ◯ Cuba
- ◯ Curacao
- ◯ Cyprus
- ◯ Czech Republic
- ◯ Denmark
- ◯ Djibouti
- ◯ Dominica
- ◯ Dominican Republic
- ◯ East Timor (see Timor-Leste)
- ◯ Ecuador
- ◯ Egypt
- ◯ El Salvador
- ◯ Equatorial Guinea
- ◯ Eritrea
- ◯ Estonia
- ◯ Ethiopia
- ◯ Fiji
- ◯ Finland
- ◯ France
- ◯ Gabon
- ◯ The
- ◯ Georgia
- ◯ Germany
- ◯ Ghana
- ◯ Greece

◯ Grenada
◯ Guatemala
◯ Guinea
◯ Guinea-Bissau
◯ Guyana
◯ Haiti
◯ Holy See
◯ Honduras
◯ Hong Kong
◯ Hungary
◯ Iceland
◯ India
◯ Indonesia
◯ Iran
◯ Iraq
◯ Ireland
◯ Israel
◯ Italy
◯ Jamaica
◯ Japan
◯ Jordan
◯ Kazakhstan
◯ Kenya
◯ Kiribati
◯ South
◯ Kosovo
◯ Kuwait
◯ Kyrgyzstan
◯ Laos
◯ Latvia
◯ Lebanon
◯ Lesotho
◯ Liberia
◯ Libya
◯ Liechtenstein
◯ Lithuania
◯ Luxembourg
◯ Macau
◯ Macedonia
◯ Madagascar
◯ Malawi
◯ Malaysia
◯ Maldives
◯ Mali
◯ Malta
◯ Marshall Islands
◯ Mauritania
◯ Mauritius
◯ Mexico
◯ Micronesia
◯ Moldova
◯ Monaco
◯ Mongolia
◯ Montenegro
◯ Morocco
◯ Mozambique
◯ Namibia
◯ Nauru
◯ Nepal
◯ Netherlands
◯ Netherlands Antilles
◯ New Zealand
◯ Nicaragua
◯ Niger
◯ Nigeria
◯ North Korea
◯ Norway
◯ Oman
◯ Pakistan
◯ Palau
◯ Palestinian Territories

◯ Panama
◯ Papua New Guinea
◯ Paraguay
◯ Peru
◯ Philippines
◯ Poland
◯ Portugal
◯ Qatar
◯ Romania
◯ Russia
◯ Rwanda
◯ Saint Kitts and Nevis
◯ Saint Lucia
◯ Saint Vincent and the Grenadines
◯ Samoa
◯ San Marino
◯ Sao Tome and Principe
◯ Saudi Arabia
◯ Senegal
◯ Serbia
◯ Seychelles
◯ Sierra Leone
◯ Singapore
◯ Sint Maarten
◯ Slovakia
◯ Slovenia
◯ Solomon Islands
◯ Somalia
◯ South Africa
◯ South Korea
◯ South Sudan
◯ Spain
◯ Sri Lanka
◯ Sudan
◯ Suriname
◯ Swaziland
◯ Sweden
◯ Switzerland
◯ Syria
◯ Taiwan
◯ Tajikistan
◯ Tanzania
◯ Thailand
◯ Timor-Leste
◯ Togo
◯ Tonga
◯ Trinidad and Tobago
◯ Tunisia
◯ Turkey
◯ Turkmenistan
◯ Tuvalu
◯ Uganda
◯ Ukraine
◯ United Arab Emirates
◯ United Kingdom
◯ United States of America
◯ Uruguay
◯ Uzbekistan
◯ Vanuatu
◯ Venezuela
◯ Vietnam
◯ Yemen
◯ Zambia
◯ Zimbabwe

6) If you reside in the United States, which State do you currently reside?

- ◯ Alabama
- ◯ Alaska
- ◯ American Samoa
- ◯ Arizona
- ◯ Arkansas
- ◯ California
- ◯ Colorado
- ◯ Connecticut
- ◯ Delaware
- ◯ District of Columbia
- ◯ Florida
- ◯ Georgia
- ◯ Guam
- ◯ Hawaii
- ◯ Idaho
- ◯ Illinois
- ◯ Indiana
- ◯ Iowa
- ◯ Kansas
- ◯ Kentucky
- ◯ Louisiana
- ◯ Maine
- ◯ Maryland
- ◯ Massachusetts
- ◯ Michigan
- ◯ Minnesota
- ◯ Mississippi
- ◯ Missouri
- ◯ Montana
- ◯ Nebraska
- ◯ Nevada
- ◯ New Hampshire
- ◯ New Jersey
- ◯ New Mexico
- ◯ New York
- ◯ North Carolina
- ◯ North Dakota
- ◯ Northern Marianas Islands
- ◯ Ohio
- ◯ Oklahoma
- ◯ Oregon
- ◯ Pennsylvania
- ◯ Puerto Rico
- ◯ Rhode Island
- ◯ South Carolina
- ◯ South Dakota
- ◯ Tennessee
- ◯ Texas
- ◯ Utah
- ◯ Vermont
- ◯ Virginia
- ◯ Virgin Islands
- ◯ Washington
- ◯ West Virginia
- ◯ Wisconsin
- ◯ Wyoming

7) Did you have COVID-19, or do you currently have it?

- ◯ Yes, confirmed by a lab test
- ◯ Yes, my health care provider told me I probably have it, but without a lab test
- ◯ I think I had or currently have COVID-19, but a health care provider did not confirm it
- ◯ No, I do not think I had it or currently have it

8) What type of confinement did you experience during the first month of COVID-19 quarantine?

- ◯ Total lockdown. (Staying at home, leaving only for basic necessities. No outdoor physical activities were permitted)
- ◯ Moderate lockdown. (Staying at home and leaving for basic necessities. Physical activities outdoors were allowed while respecting social distancing)
- ◯ Light lockdown. (No home confinement. No measures were applied other than social distancing)
- ◯ Other

9) How many weeks have you been quarantined?

____

10) What is the preferred video game genre that you currently play or compete in the most. If unsure, please write the genre or game under others.

- ☐ Massive Multiplayer Online Role Playing Game (MMORPG)
- ☐ First Person Shooter (FPS)
- ☐ Multiplayer Online Battle Arena (MOBA)
- ☐ Digital Collectible Card game (DCCG)
- ☐ Battle Royale
- ☐ Sports game
- ☐ Fighting
- ☐ Racing
- ☐ Others

11) Presently, what is your highest level of gaming competition?

- ☐ Recreational (Esports is a leisure activity not financially compensated)
- ☐ Amateur (Esports is a leisure activity with occasional small financial compensation)
- ☐ High school (I compete in Esports in a High school environment)
- ☐ College (I compete in Esports in a College environment)
- ☐ Professional (Esports is my full-time occupation)

12) Prior to COVID-19, On average how many days per week did you Game?

- ◯ 1
- ◯ 2
- ◯ 3
- ◯ 4
- ◯ 5
- ◯ 6
- ◯ 7

13) During COVID-19 quarantine, on average how many days per week did you game?

- ◯ 0
- ◯ 1
- ◯ 2
- ◯ 3
- ◯ 4
- ◯ 5
- ◯ 6
- ◯ 7

14) Prior to COVID-19, on average how many hours per day did you game?

- ◯ 0-1h
- ◯ 1-3h
- ◯ 3-5h
- ◯ 5-7h
- ◯ 7-9h
- ◯ 9-11h
- ◯ 11+

15) During COVID-19 quarantine, on average how many hours per day did you game?

- ◯ 0-1h
- ◯ 1-3h
- ◯ 3-5h
- ◯ 5-7h
- ◯ 7-9h
- ◯ 9-11h
- ◯ 11+

16) At what time of the day do you most often game? (You can choose multiple options)

- ☐ Early morning (5am - 9am)
- ☐ Late morning (9am - 12pm)
- ☐ Early afternoon (12pm - 3pm)
- ☐ Late afternoon (3pm - 6pm)
- ☐ Evening (6pm - 9pm)
- ☐ Early night (9pm -12am)
- ☐ Late night (12 am - 5am)

17) At what time do you feel you perform your best in-game?

- ◯ Early morning (5am - 9am)
- ◯ Late morning (9am - 12pm)
- ◯ Early afternoon (12pm - 3pm)
- ◯ Late afternoon (3pm - 6pm)
- ◯ Evening (6pm - 9pm)
- ◯ Early night (9pm -12am)
- ◯ Late night (12 am - 5am)

18) Prior to the COVID-19 quarantine, on average, how many days per week did you engage in moderate and/or vigorous physical activity?

- ◯ 0
- ◯ 1
- ◯ 2
- ◯ 3
- ◯ 4
- ◯ 5
- ◯ 6
- ◯ 7

19) During COVID-19 quarantine, on average, how many days per week did you engage in moderate and/or vigorous physical activity?

- ◯ 1
- ◯ 2
- ◯ 3
- ◯ 4
- ◯ 5
- ◯ 6
- ◯ 7

20) Prior to the COVID-19 quarantine, how long did your exercise sessions last?

- ◯ < 30 min
- ◯ 30 min - 1 hour
- ◯ 1-2 hours
- ◯ 2-3 hours
- ◯ 3+ hours

21) During COVID-19, how long did your exercise sessions last?

- ◯ < 30 min
- ◯ 30 min - 1 hour
- ◯ 1-2 hours
- ◯ 2-3 hours
- ◯ 3+ hours

22) Prior to the COVID-19 quarantine, how much time did you spend sitting every day (overall, including gaming)?

- ◯ 0-4 hours
- ◯ 4-6 hours
- ◯ 6-8 hours
- ◯ 8-10 hours
- ◯ 10-12 hours
- ◯ 12-14 hours
- ◯ 14-16 hours
- ◯ 16+ hours

23) During COVID-19 quarantine, how much time did you spend sitting every day (overall, including gaming)?

- ◯ 0-4 hours
- ◯ 4-6 hours
- ◯ 6-8 hours
- ◯ 8-10 hours
- ◯ 10-12 hours
- ◯ 12-14 hours
- ◯ 14-16 hours
- ◯ 16+ hours

24) Indicate up to 3 biggest barriers that prevent you from engaging in physical activity

- ☐ Lack of time
- ☐ Lack of money
- ☐ Lack of motivation
- ☐ Lack of interest
- ☐ I have no barriers that prevent me from engaging in physical activity
- ☐ Other

25) Prior to Covid-19 how satisfied were you with your nutrition on a regular basis?

- ◯ Very satisfied
- ◯ Somewhat satisfied
- ◯ Neither satisfied nor dissatisfied
- ◯ Somewhat dissatisfied
- ◯ Very dissatisfied

26) During COVID-19 quarantine, how satisfied were you with your nutrition on a regular basis?

- ◯ Very satisfied
- ◯ Somewhat satisfied
- ◯ Neither satisfied nor dissatisfied
- ◯ Somewhat dissatisfied
- ◯ Very dissatisfied

27) Indicate up to 3 of the biggest barriers that prevent you from eating healthier.

- ☐ Lack of time
- ☐ Lack of money
- ☐ Lack of motivation
- ☐ Lack of Interest
- ☐ I have no barriers that prevent me from eating healthy
- ☐ Other

28) Prior to the COVID-19 quarantine, on average, how many hours of actual sleep did you get per night? (This may be different than the number of hours you spent in bed).

- ◯ < 5 hours
- ◯ 5-6 hours
- ◯ 6-7 hours
- ◯ 7-8 hours
- ◯ 8-9 hours
- ◯ >9 hours

29) During COVID-19 quarantine, on average, how many hours of actual sleep did you get per night? (This may be different than the number of hours you spent in bed).

- ◯ < 5 hours
- ◯ 5-6 hours
- ◯ 6-7 hours
- ◯ 7-8 hours
- ◯ 8-9 hours
- ◯ >9 hours

30) Prior to the COVID-19 quarantine, how satisfied were you with the quality of your sleep?

◯ Very satisfied
◯ Somewhat satisfied
◯ Neither satisfied nor dissatisfied
◯ Somewhat dissatisfied
◯ Very dissatisfied

31) During COVID-19 quarantine, how satisfied were you with the quality of your sleep?

◯ Very satisfied
◯ Somewhat satisfied
◯ Neither satisfied nor dissatisfied
◯ Somewhat dissatisfied
◯ Very dissatisfied

32) Indicate up to 3 biggest barriers that prevent you from sleeping better

☐ Lack of time
☐ Excessive noise
☐ Excessive light
☐ Uncomfortable sleeping space
☐ Stress
☐ Physical discomfort
☐ Too high or low room temperature
☐ I have no barriers. My sleep is fine.
☐ Other

33) Prior to the COVID-19 quarantine, how were your perceived levels of stress and anxiety?

No levels of stress | Moderate levels of stress | Maximum levels of stress

(Place a mark on the scale above)

34) During COVID-19 quarantine, how were your perceived levels of stress and anxiety?

No levels of stress | Moderate levels of stress | Maximum levels of stress

(Place a mark on the scale above)
